# Immersive Learning With AI‐Enhanced Virtual Dental Standardized Patient

**DOI:** 10.1002/jdd.13994

**Published:** 2025-07-16

**Authors:** Betti Shahin, Judy Chia‐Chun Yuan, Markus Santoso, Cortino Sukotjo

**Affiliations:** ^1^ Department of Restorative Dentistry College of Dentistry University of Illinois Chicago Chicago Illinois USA; ^2^ Digital Worlds Institute University of Florida Gainesville Florida USA; ^3^ Department of Prosthodontics School of Dental Medicine University of Pittsburgh Pittsburgh Pennsylvania USA

**Keywords:** Computer Simulation, Education, Educational Technology, Patient Affairs, Patient Simulation, Patient‐Provider Interaction, Technology

## 1 Problem

The actor‐based standardized patient (SP) has been integrated into health care education to promote medical history taking and improve the communication skills of the students [1, 2]. While SPs provide the learners an effective way to establish strong communication skills with a patient and real‐time standardized feedback, there may be challenges in providing consistent and repetitive training. Virtual standardized patient (VSP) has been widely used in medical education to address some deficiencies of the SP approach [3, 4]. VSP enables learners to practice history taking, develop differential diagnoses, and enhance empathy before encounters with real patients [3, 4]. VSP may provide students a controllable, secure, and safe learning environment with the opportunity for extensive repetitive practice with feedback. However, the current technology used for VSP in medical education is still site and time‐dependent, and not as cost‐effective. Further, limited VSP specifically for dental education has been developed to date.

## 2 Solution

**FIGURE 1:**
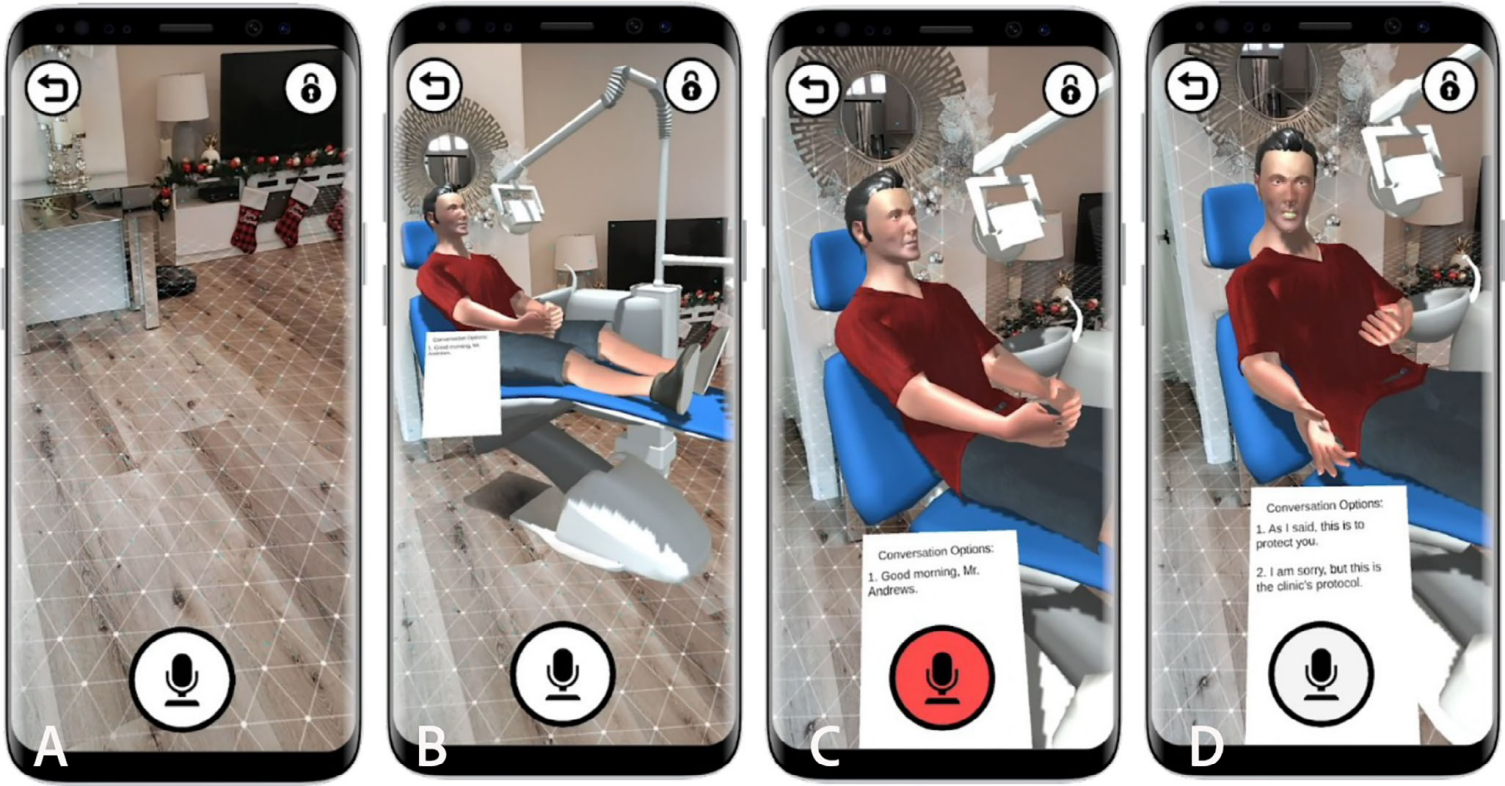
The augmented reality mode allows users to scan the environment (A), position the virtual patient in the designated location (B), and initiate interaction with the avatar (C and D).

**FIGURE 2:**
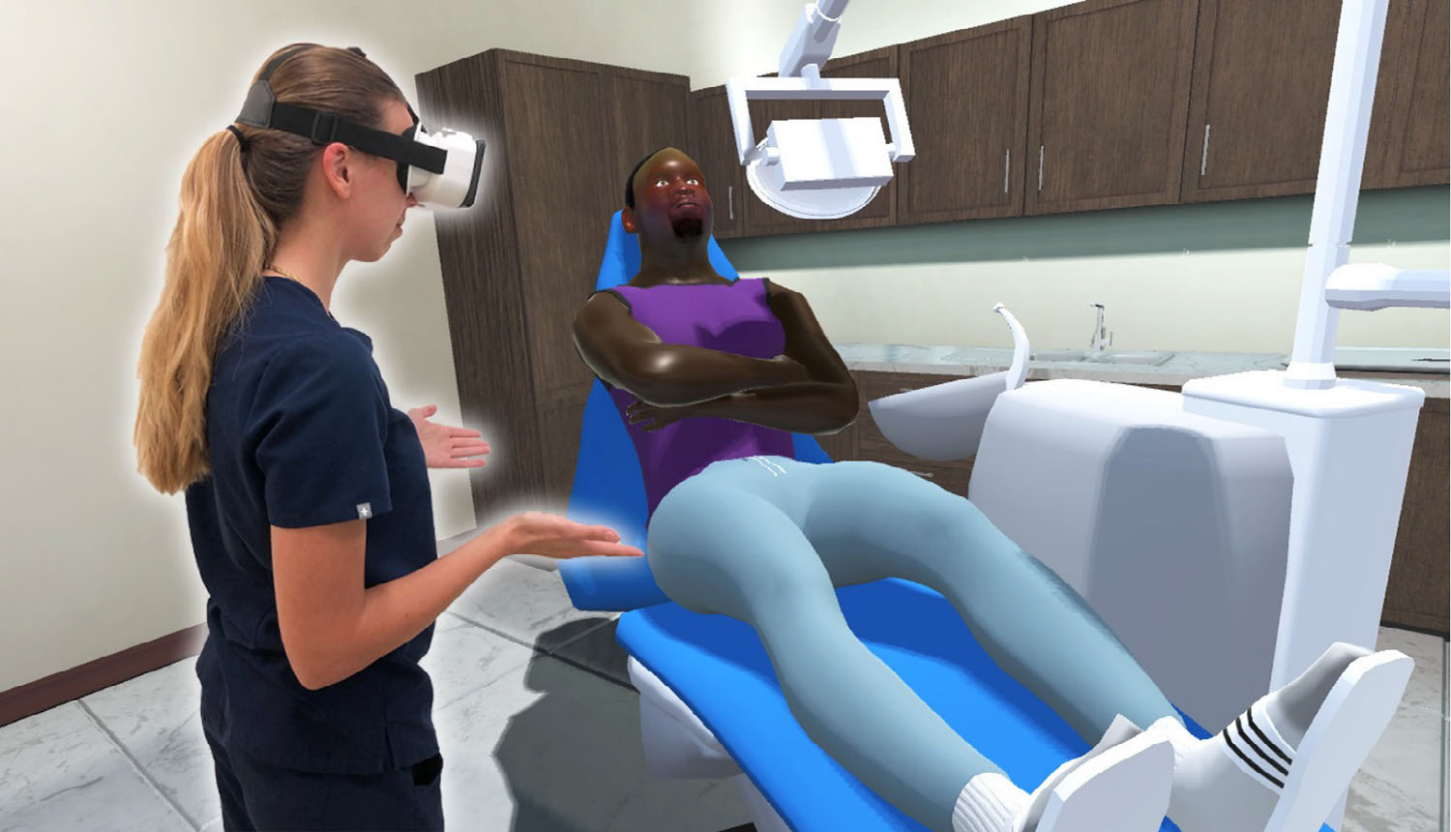
The fully immersive mode allows students to engage in a virtual dental clinic environment and begin interacting with the avatar.

The authors developed a mobile, cost‐effective, artificial intelligence (AI)‐enhanced virtual dental standardized patient (VDSP) to enhance dental students’ communication skills. The AI‐enhanced VSP uses natural language processing to facilitate seamless communication between the learner and the virtual patient. This project also adds an adaptive storytelling approach controlled with AI to allow learners to explore different conversation flows and learn various patient responses. This research employed an augmented reality (AR) technology to immerse the virtual patient in the user's space. Using the AR feature, the learners would be able to scan their room, place the virtual patient on the assigned spot, and communicate with the avatar (Figure 1). In this application, an option for the learners to switch from AR mode to the full‐immersed mode (Figure 2) where the learners can slide their phone onto the VR viewer, such as Google Cardboard, was provided. In the fully immersive mode, learners engage with an avatar in a realistic virtual dental clinic, simulating real‐world interactions. To measure the acceptability and usability of the VDSP, fourth‐year predoctoral dental students at the University of Illinois Chicago, College of Dentistry, who had prior experience with the SP through their third‐year classroom lectures and objective structured clinical examination, were invited to participate. Students were invited to test both applications (AR and full‐immersed modes) for 30 min, during which they conducted a simulated initial examination by collecting medical and dental history through conversation with the virtual avatar. Immediately after the virtual scenario, students were invited to complete a modified, validated technology‐related SP questionnaire [5]. Several domains were investigated, including the usability of the application, the realistic and authentic clinical scenario, and the perception of communicating with VDSP compared with SP.

## 3 Results

Ninety‐eight students participated in the study (Table 1). Overall, participants rated most of the survey items highly (≥4), indicating a generally positive response. Most students reported familiarity with the SP method and expressed comfort in using digital tools. However, fewer students were familiar with the concept of VDSP. Participants agreed that the VDSP's intonation, tone, and voice were realistic, and many believed these features could enhance both their motivation and communication skills. They also appreciated the 24/7 accessibility of the VDSP platform and recognized its potential to improve communication, particularly in challenging interactions such as managing dissatisfied patients. At the same time, students acknowledged limitations of the current avatar system, noting that it was not yet fully realistic or authentic and required further improvements to appear more human‐like. Despite these limitations, there was strong agreement that the VDSP aligns with the growing emphasis on digital learning and should be integrated into the dental curriculum.

**TABLE 1:** Assessment of the virtual dental standardized patient (VDSP).

No.	Questions	Response (*n* = 98)
1	I am familiar with the “standardized patient” simulating learning method.	Yes: 92, No: 6
2	I am familiar with the concept of the “virtual standardized patient” learning method.	Yes: 79, No: 19
3	I am comfortable working with gadgets and computer.	Yes: 94, No: 4
4	The software was user‐friendly.	4.16 ± 0.85
5	I needed multiple technical assistances.	2.42 ± 1.19
6	The interaction with the VDSP was practical.	4.23 ± 0.84
7	I prefer the VDSP experience more than the SP experience.	3.76 ± 1
8	The overall experience with VDSP felt real.	4.07 ± 0.87
9	The amount of time allocated for training was adequate.	4.07 ± 0.83
10	The training was repeatable.	4.31 ± 0.76
11	The VDSP experience enhanced my motivation.	4.05 ± 0.89
12	The VDSP experience enhanced my communicational skill and made me ask questions.	4.23 ± 0.78
13	The VDSP experience enhanced how to communicate with a dissatisfied patient.	4.37 ± 0.62
14	The VDSP learning method should be added to the preclinical dental curriculum.	4.37 ± 0.66
15	The VDSP learning method should be added to the clinical dental curriculum to practice my communication skills.	4.37 ± 0.71
16	The VDSP learning does comply with ever‐expanding digital learning worldwide.	4.5 ± 0.69
17	I had a sense of “being there” in the virtual exam room.	4.1 ± 0.88
18	The importance of the VDSP being life‐sized.	3.99 ± 0.88
19	The interaction of the virtual patient was authentic.	4.0 ± 0.86
20	The VDSP gestures were life‐like.	3.98 ± 0.9
21	The VDSP appears authentic.	3.89 ± 0.9
23	The intonation/tones were life‐like.	4.26 ± 0.7
24	The quality of the voice of the virtual patient was good.	4.37 ± 0.67
25	Compared to traditional SP, VDSP is more available (no time and location boundaries).	4.5 ± 0.6
26	Compared to traditional SP, VDSP follows more the current trend (futuristics).	4.41 ± 0.62
27	Compared to traditional SP, VDSP is more repeatable.	4.45 ± 0.66
28	Compared to traditional SP, VDSP is more realistic.	3.06 ± 1.2

*Note*: Strongly disagree = 1 to strongly agree = 5.

## 4 Lessons Learned

The proposed project applied advanced technology as an innovative teaching tool and blends digital elements with an authentic learning environment. Student perceptions highlighted the significant promise of the integration of dental VDSP in dental education. Further enhancements are necessary to increase the avatar's realism, making it appear more humanoid and lifelike in both facial expressiveness and human‐like motion. This new teaching possibility has positively affected the quality of learning and promoted interactive learning in patient communication and management. VDSP simulations can reduce the expense, faculty time, and resources needed to aid the students in developing and enhancing their patient‐centered communication skills.
